# Machine learning-based models for predicting mortality and acute kidney injury in critical pulmonary embolism

**DOI:** 10.1186/s12872-023-03363-z

**Published:** 2023-08-02

**Authors:** Geng Wang, Jiatang Xu, Xixia Lin, Weijie Lai, Lin Lv, Senyi Peng, Kechen Li, Mingli Luo, Jiale Chen, Dongxi Zhu, Xiong Chen, Chen Yao, Shaoxu Wu, Kai Huang

**Affiliations:** 1 Department of Vascular Interventional Radiology, Zhongshan Hospital of Traditional Chinese Medicine, Zhongshan, China; 2 Department of Cardiovascular Surgery, Sun Yat-Sen Memorial Hospital, Sun Yat-Sen University, No.33, Yingfeng Road, Haizhu District, Guangdong Province 510000 Guangzhou, China; 3 Zhongshan School of Medicine, Sun Yat-Sen University, Guangzhou, China; 4 Department of Medicine, Sun Yat-Sen Memorial Hospital South Campus Clinic, Guangzhou, China; 5 Hospital of Stomatology, Guanghua School of Stomatology, Sun Yat-Sen University, Guangzhou, China; 6 Department of Urology, SunYat-Sen Memorial Hospital, SunYat-Sen University, Guangzhou, China; 7 Department of Vascular Surgery, First Affiliated Hospital of Sun Yat-Sen University, Guangzhou, China; 8 Guangdong Provincial Key Laboratory of Malignant Tumor Epigenetics and Gene Regulation, Guangzhou, China

**Keywords:** Pulmonary embolism (PE), Machine learning (ML), Prognosis, Mortality, Intensive care unit

## Abstract

**Objectives:**

We aimed to use machine learning (ML) algorithms to risk stratify the prognosis of critical pulmonary embolism (PE).

**Material and methods:**

In total, 1229 patients were obtained from MIMIC-IV database. Main outcomes were set as all-cause mortality within 30 days. Logistic regression (LR) and simplified eXtreme gradient boosting (XGBoost) were applied for model constructions. We chose the final models based on their matching degree with data. To simplify the model and increase its usefulness, finally simplified models were built based on the most important 8 variables. Discrimination and calibration were exploited to evaluate the prediction ability. We stratified the risk groups based on risk estimate deciles.

**Results:**

The simplified XGB model performed better in model discrimination, which AUC were 0.82 (95% CI: 0.78–0.87) in the validation cohort, compared with the AUC of simplified LR model (0.75 [95% CI: 0.69—0.80]). And XGB performed better than sPESI in the validation cohort. A new risk-classification based on XGB could accurately predict low-risk of mortality, and had high consistency with acknowledged risk scores.

**Conclusions:**

ML models can accurately predict the 30-day mortality of critical PE patients, which could further be used to reduce the burden of ICU stay, decrease the mortality and improve the quality of life for critical PE patients.

**Supplementary Information:**

The online version contains supplementary material available at 10.1186/s12872-023-03363-z.

## Introduction

Pulmonary embolism (PE) is a clinical manifestation of venous thromboembolism (VTE) and is the third most common cause of cardiovascular death worldwide after stroke and heart attack [1]. In the United States, PE killed 300,000 people per year [2]. PE is usually caused by deep vein thrombosis (DVT) of the lower extremity. And the clinical manifestations can vary from asymptomatic to fatal [3]. Although there are a number of auxiliary examinations (such as computed tomography pulmonary angiogram [CTPA], echocardiography, etc.) that can help us identify the serious condition, their effects do not seem to be obvious [4]. Thus, sorting out the patients with acute PE remains a big challenge. There are currently clear relevant studies on the risk grading of PE, including the European Society of Cardiology (ESC), Australia and New Zealand Risk of Death (ANZROD), simplified pulmonary embolism severity index (sPESI), pulmonary embolism severity index (PESI) and so on [5–9]. However, whether these scores could also be applied in intensive care unit (ICU) still remains unknown. ICU is the best place to monitor and support critical-ill PE patients [10] but there is no relevant definition of disease heterogeneity in PE patients in ICU and no clear guidelines to recommend how we should manage them individually. As a result, it is difficult for physicians in ICU to grade risk of patients.

Critical-ill PE patients are usually characterized by adverse complications (such as acute kidney injury [AKI], predominantly malignancy, etc.), requirement of mechanical ventilation (MV), trend of hemodynamic instability, and high mortality [11–14]. Although the pathophysiology is not well understood, PE has been proved to be a unique cause of AKI [14]. We supposed that some prediction methods can be used to stratify risk of PE patients, by which we can identify low-risk patients and allow them to discharge from ICU early. For high-risk patients, we should not only prevent death, but also prevent the occurrence of AKI. In this way, we can not only reduce the resource burden of ICU for healthcare centers and the unnecessary burden of ICU stay for patients, but also manage patients with critical PE more effectively and improve their survival rate and quality of lives [12].

Machine learning (ML) algorithms, which uses computers to learn data and capture high-dimensional, non-linear relationships between clinical features to make data-driven outcome predictions, has been widely accepted in the medical field [15, 16]. We used ML to construct models to predict the outcomes of in-ICU death, in-Hospital death, and AKI in patients with critical PE. The data was obtained from Medical Information Mart for Intensive Care IV (MIMIC-IV version 1.0) database [17]. The models included logistic regression (LR), simplified eXtreme gradient boosting (XGBoost) [18]. We selected the final model after comparing their matching degree and prediction ability with the data.

## Material and methods

### Patients and materials

The data we used to developed and validated the model was derived from the MIMIC-IV database from 2008 to 2019 and 1229 patients were included, of whom 860 patients (70%) were assigned into the derivation cohort and 369 (30%) were assigned into the validation cohort. MIMIC-IV database is a large, freely-available ICU database involving clinical data in Beth Israel Deaconess Medical Center, which extracts patient data from hospitals and ICU, corresponds it to medical record numbers, then reorganizes the data, simplifies the database, and performs patient identification with high accuracy and simplicity [17]. International classification of diseases (ICD)-9 or -10 version diagnostic code was used to define the patients’ disease condition in MIMIC-IV database. Critical PE patients with/without septic or other cardiopulmonary complications were all included in this analysis, which was diagnosed based on ICD-9 codes of 41,511, 41,512, ICD-10 codes of I26, I260, I2601, I2609, I269, I2690, I2693 and I2699. The diagnosis and clinical treatment flow could be referred to the 2019 ESC guideline for the diagnosis and management of critical PE. CTPA with clinical manifestation including dyspnea, chest pain, presyncope or hemoptysis was the golden diagnoses criterion. For risk stratification, sPESI, 2019 ESC PE risk stratification model, simplified acute physiology score II (SAPSII) and sequential organ failure assessment (SOFA) were used to evaluate the risk for every patient. For ESC model, hemodynamic decompensation included cardiac arrest or need for cardiopulmonary resuscitation, obstructive shock (including systolic blood pressure [SBP] < 90 mmHg or vasopressors required, and end-organ hypoperfusion) and persistent hypotension (including SBP < 90 mmHg or SBP drop > 40 mmHg, lasting longer than 15 min and not caused by new-onset arrhythmia, hypovolemia, or sepsis). The exclusion criteria included age < 18, not first ICU admission or hospital admission, and not emergence admission (elective admission and observation admission were excluded).

### Clinical treatment

For critical PE patients, treatment included hemodynamic and respiratory support, initial anticoagulation, reperfusion treatment, vena cava filters, etc. Oxygen therapy was indicated in patients with SpO2 < 90%. High-flow oxygen and mechanical ventilation (non-invasive or invasive) were used in a worse situation. In cardiac arrest presumably caused by acute PE, current guidelines for advanced life support should be followed. In the acute phase of high-risk PE, systemic thrombolytic therapy and immediate anticoagulation with unfractionated heparin (UFH) were recommended. Vasopressor was also an important treatment in hemodynamic decompensation especially congestive heart failure or cardiogenic shock and it included using dobutamine, dopamine, epinephrine, norepinephrine, phenylephrine or milrinone here. When thrombolysis was contraindicated or failed, surgical pulmonary embolectomy or percutaneous catheter-directed treatment was recommended. Anticoagulation should be initiated immediately in patients with a high or intermediate clinical probability of PE. Inferior vena cava (IVC) filters should be considered in patients with acute PE, who had absolute contraindications to anticoagulation or in cases of PE recurrence despite therapeutic anticoagulation.

### Outcomes and variables definition

From MIMIC-IV database, we obtained general information, laboratory test, vital signs, complications, treatment information and severity scores. Laboratory results were all tested within 24 h after ICU admission, including cardiac markers, hematological parameters, biochemical markers and coagulative markers. Vital signs included blood pressure, heart rate, respiratory rate, SpO2 and temperature tested within 24 h after ICU admission. For treatment information, oxygen therapy included high flow nasal cannula oxygen inhalation and mechanical ventilation (non-invasive or invasive). Urine output was the total volume within the 24 h after ICU admission. The invasive line included both arterial and venous catheter (also included dialysis catheters).

Primary outcomes were set as all-cause mortality within 30 days. AKI was defined based on Kidney Disease Improving Global Outcomes (KDIGO) and was determined by serum creatinine (SCr) and urine output (reduced urine output [urine volume < 0.5 mL/kg/h for ≥ 6 h] and increased SCr level [an increase in SCr of ≥ 0.3 mg/dL within 48 h or an increase in SCr to ≥ 1.5 times baseline within 7 days]).

### Imputation of missing value

Only variables with missing value proportion less than 20% were put into constructing prediction models by ML algorithms (Supplementary Table 1). R package “MICE” was used to impute the missing value, based on the complete conditional specification and predictive mean matching method. Each missing variable was imputed using an independent model to ensure the validity. To ensure the authenticity of the risk prediction sores, sPESI, ESC model, SAPSII and SOFA scores were not imputed. The missing outcomes were also not imputed.

### Feature selection and model development

Four general baseline variables included gender, age, proximal DVT and VTE history. Maximum and minimum of 15 laboratory variables (hematocrit, hemoglobin, anion gap, e.g.) within 24 h after ICU admission, maximum and minimum of 8 vital signs (heart rate, blood pressure, respiratory rate, e.g.) within 24 h after ICU admission, 22 variables of complications (congestive heart failure, hypertension, atrial fibrillation, e.g.), risk status of hemodynamic instability (including one treatment variable of vasopressor use) and 3 supporting treatment variables included ventilation, urine output and invasive line, were used for feature selection, model development and prediction for the main outcome (More details are shown in Supplementary Table 1).

In order to balance the number of positive and negative examples in the derivation cohort to overcome overfitting, the synthetic minority over-sampling technique (SMOTE) was applied to synthesize new samples and add them to the derivation cohort. Then, zero-mean normalization of continuous variables was conducted to reduce the relevant impact of non-normality on the model performance.

XGBoost has been regarded to perform well in predicting binary classification of outcome, while LR has high interpretability by weighting of the features of models [19, 20]. Considering that primary outcome is binary, ML algorithms comprising XGB and LR were implemented for model constructions, and ROC curve was also constructed to evaluate the discrimination of our models. And we then used the grid search method to optimize the hyperparameters. The relative importance of clinical variables in each model was determined based on the effect on outcomes, which was then ranked and shown as radar plots. After building full-variables models, 8 relatively more important variables were chosen to build simplified models for further clinical use.

### Evaluation and validation of the model

The most important indicators to evaluate the prediction performance were discrimination and calibration, which were usually based on AUC and calibration curves. Firstly, the derivation cohort was separated into the training cohort and internal validation cohort to conduct tenfold cross validation to investigate the stability. Then the models were validated in the validation cohort to evaluate the generalization. After calculating the best cut-off value based on receiver operator curve (ROC) and Youden index, ML indicators involving F scores, precision recall, false accept rate (FAR), positive prediction value, negative prediction value, accuracy, sensitivity and specificity were calculated. sPESI was also externally validated. Net reclassification improvement (NRI) and integrated discrimination improvement (IDI) were used to compare our ML models to sPESI on the prediction performance. To characterize the crucial characteristics that affect mortality risk in ML model, we plotted bar graph consisting of average SHAP value for each feature.

### Risk classification

Risk classification is not only for convenient use in clinical, but also an important part of the calibration. Patients in each dataset were divided into estimated risk deciles in accordance with predictive outputs of ML models. We then calculated the mean prediction probability and observed probability in each group, and observed the calibration result in each decile group. Patients were divided into low-, intermediate-, and high-risk groups based on thresholds that highlight significant gradients in risk from one relative lower risk group to the next higher group in risk deciles plots and the calibration plots. Finally, we compared the clinical outcomes and risk scores in each group to investigate whether the new risk classification systems could reflect real-world risk of critical PE patients. Furthermore, we performed decision curve analysis (DCA) on XGBoost model to determine whether the model can improve clinical decision making.

### Statistical analysis

When comparing the baseline data and clinical outcomes in the derivation and validation cohorts, categorical variables were expressed as percentages, compared using chi-square tests, while continuous variables were presented by median with interquartile range (IQR) and compared using Kruskal–Wallis test. A two-sided *P* < 0.05 was defined statistically significant. Data imputation, cleaning and transforming were implemented in R (version 3.6.3). Variables selection, model constructions, performance evaluation and validation were carried out in Python (version 3.8.5). Data pre-processing and Logistic regression model development were conducted with scikit-learn library. XGBoost models were developed to select features and validate using xgboost package of Python. Shapley additive explanations (SHAP) values were calculated by the SHAP package of Python. SMOTE was conducted using imblearn package. The figures were drawn by matplotlib.pyplot library.

### Ethics

The study was conducted in accordance with the Declaration of Helsinki, and approved by the Institutional Review Board (or Ethics Committee) of both Beth Israel Deaconess Medical Center and Massachusetts Institute of Technology Affiliates (protocol code 35,655,780 and 03-Mar-2020), Sun Yat-sen Memorial Hospital (SYSKY-2023–199-01). Requirement for patient consent was waived because this was a retrospective study and did not impact clinical practice.

## Results

After our filtering, a total of 1229 patients from MIMIC-IV database were included in this study. There were 860 (70%) patients in the derivation cohort and 369 (30%) patients in the validation cohort. The derivation cohort was used to develop models with ML approaches including XGB, LR and conduct internal validation (tenfold cross validation). While the validation dataset was used to verify the efficiency and generalization of the models. When comparing baseline data in the derivation and validation cohorts, only age showed difference between them (*P* = 0.034, Table 1, Supplementary Table 1). The derivation cohort had older age. Male occupied 51% of the derivation cohort, and 48.5% of the validation cohort. Hemodynamic instability occupied 35.3% of the derivation cohort, and 34.3% of the validation cohort. 31.4% of the derivation cohort and 30.9% of the validation cohort received invasive mechanical ventilation. Variables included in model development are shown in Supplementary Table 1. To further ensure the balance between the derivation cohort and the validation cohort, survival curves of each group were drawn, and the results showed no difference in survival time between the two groups (Log rank *P* = 0.28, Supplementary Fig. 1).

**Table 1:** Baseline data and clinical outcomes for derivation and validation cohort

Baseline Data	Derivation Cohort (*N* = 860)	Validation Cohort (*N* = 369)	*P* Value
Male	439 (51.0%)	179 (48.5%)	0.451
Age	67.46 [55.88, 78.93]	65.86 [54.61, 75.67]	0.034
BMI	28.00 [23.90, 33.15]	29.70 [25.35, 34.90]	0.010
Admission Type			0.885
Emergency	647 (75.2%)	273 (74.0%)	
Surgical Admission	23 (2.7%)	11 (3.0%)	
Urgent	190 (22.1%)	85 (23.0%)	
Proximal DVT	114 (13.3%)	63 (17.1%)	0.097
VTE History	43 (5.0%)	19 (5.1%)	1
Hemodynamic Instability	304 (35.3%)	127 (34.4%)	0.804
Urine Output in First Day	1280.00 [791.25, 2043.75]	1305.00 [800.00, 2020.00]	0.75
Ventilation			0.834
None	117 (13.6%)	55 (14.9%)	
Non-Invasive	473 (55.0%)	200 (54.2%)	
Invasive	270 (31.4%)	114 (30.9%)	
Invasive Line	469 (54.5%)	208 (56.4%)	0.596
sPESI score	2.00 [1.00, 3.00]	2.00 [1.00, 3.00]	0.098
ESC model			0.937
High Risk	304 (35.5%)	127 (34.4%)	
Intermediate Risk	495 (57.8%)	217 (58.8%)	
Low Risk	58 (6.8%)	25 (6.8%)	
SAPSII	34.00 [26.00, 45.00]	33.00 [24.00, 43.00]	0.209
SOFA	4.00 [2.00, 6.00]	3.00 [1.00, 7.00]	0.757
Clinical Outcome			
In-ICU Death	83 (9.7%)	47 (12.7%)	0.131
In-Hospital Death	148 (17.2%)	70 (19.0%)	0.51
AKI	559 (65.0%)	231 (62.6%)	0.46
ICU Stay Length	2.19 [1.18, 4.87]	2.35 [1.09, 5.24]	0.957
Hospital Stay Length	8.62 [4.78, 16.04]	8.13 [4.80, 15.71]	0.627

Categorical variables are expressed as percentages, compared with chi-square tests, continuous variables were represented by the median with interquartile range (IQR) and compared using the Kruskal–Wallis test. A two-sided *P* < 0.05 was considered statistically significant

*Abbreviations*: *BMI* body mass index, *DVT* deep vein thrombosis, *VTE* venous thromboembolism, *sPESI* simplified pulmonary embolism severity index, *ESC* European society of cardiology, *SAPSII* simplified acute physiology score II, *SOFA* sequential organ failure assessment, *ICU* intensive care unit, *AKI* acute kidney injury

The relative importance in final model is shown in the radar plots **(**Supplementary Fig. 2). The top 8 important variables were chosen for building simplified models. As for XGB model, the top 8 important variables included international normalized ratio (INR, maximum and minimum), gender, sinus-tachycardia, creatinine (maximum), chronic pulmonary disease, renal disease, and history of VTE (Supplementary Fig. 2).

**Fig. 1:**
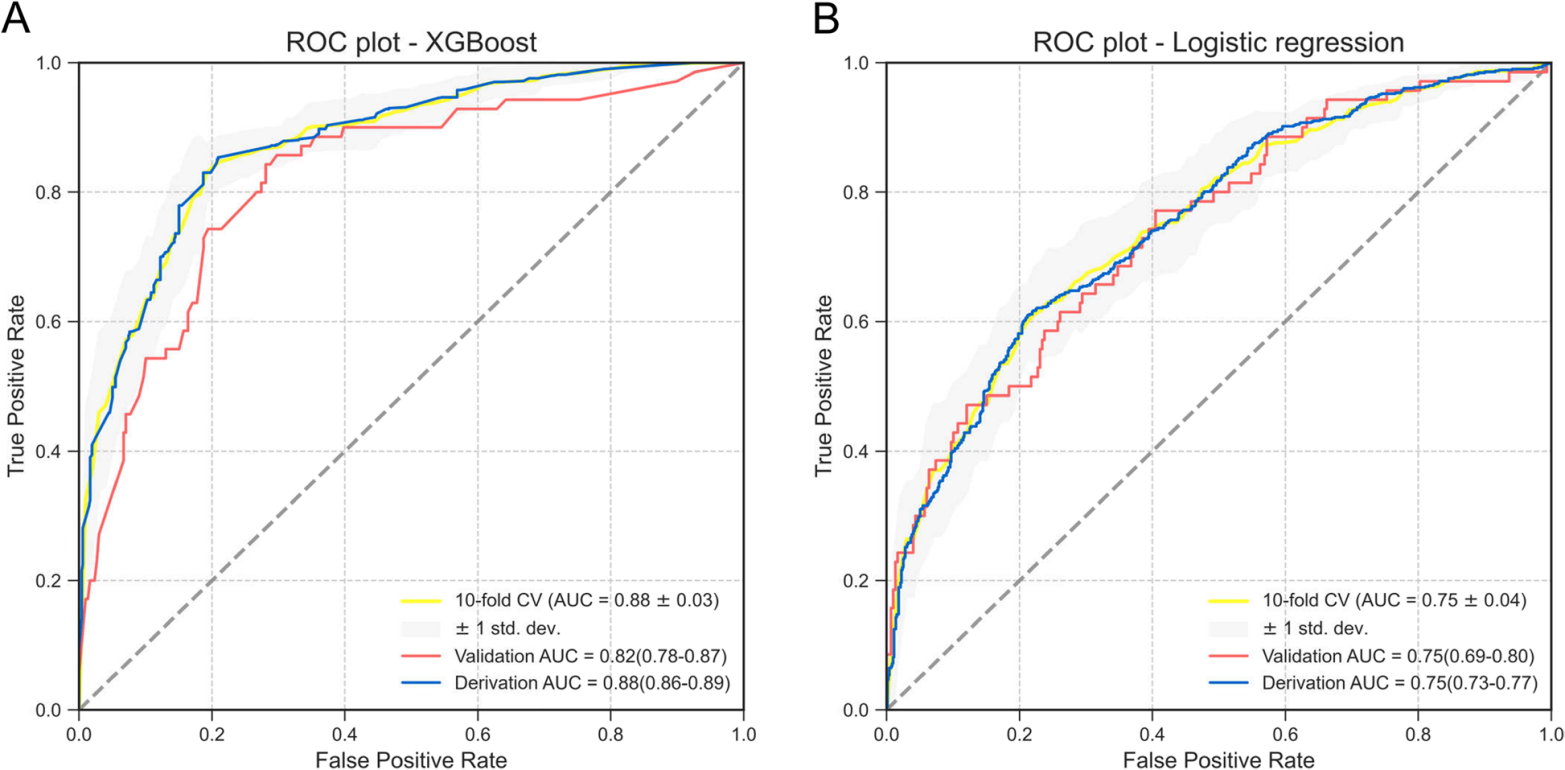
ROC curves based on ML models in derivation and validation cohort. The top 8 relative more important variables were chosen to build ML models. Receiver operating characteristic curves for primary outcomes (30-days mortality) based on XGBoost and LR models. AUC was presented as mean with 95% CI. Abbreviation: ROC, receiver operating characteristic curve; AUC, area under the curve; XGB, eXtreme gradient boosting; LR, logistic regression

**Fig. 2:**
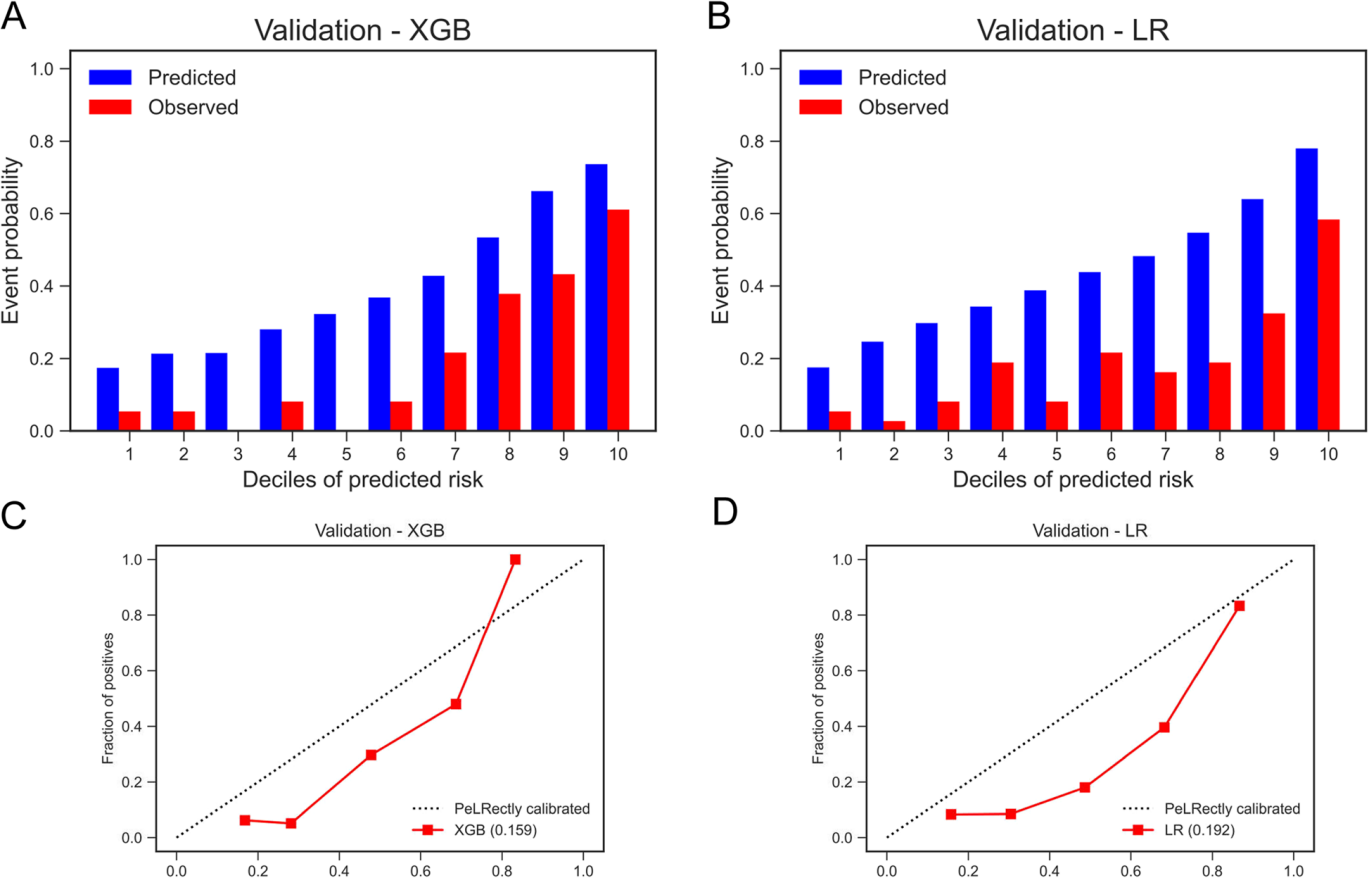
Risk of outcome and calibration curve in validation cohort based on ML models. Risk of primary outcome according to deciles of event probability based on ML models in the validation cohort. **A** XGBoost model; **B** LR model. Calibration curve shows the mean predicted probability of outcome against the observed proportion of clinical outcomes. **C** XGBoost model; **D** LR model

Building simplified models aimed to make convenience for clinical decisions, and we still found their good prediction ability in the validation cohort (Fig. 1). The simplified XGB model performed better in model discrimination, which AUC were 0.82 (95% CI: 0.78–0.87), compared with the AUC of simplified LR model (0.75 [95% CI: 0.69—0.80]), thus the simplified XGB model is considered to be more suitable for clinical application. The risk of outcomes in the validation cohort according to deciles of event probability based on simplified models is shown in Fig. 2. The observed probability tended to increase with the predicted probability. Compared with LR model, the XGB model had the smaller differences in observed probability and predicted probability in each decile group for predicting the all-cause mortality within 1 month (Fig. 2A, B). As an example of risk stratification, we divided the patients into three groups according to the predicted all-cause mortality using the XGB model. We set the first group as low-risk, which the prediction probability was lower than 20%, while second group as intermediate-risk (prediction probability of 20–40%) and high-risk group patients were set as with the prediction probability > 40%. The risk of outcomes in the derivation cohort according to deciles of event probability based on finally models are shown in Supplemental Fig. 3.

In addition, we also plotted the calibration curve based on simplified models in the validation cohort (Fig. 2C, D). The calibration curve based on final models in derivation cohort is shown in Supplemental Fig. 4. When predicting 30-days mortality, we found that simplified ML models had overestimated the event probability. As a result, patients judged as low-risk by our ML models would have even more low risk of death than predicted and therefore be safe enough. We also summarized the performance metrics of our models in validation cohort, and the results showed that negative predictive value (NPV) was up to 0.904 (Table 2), which also indicates that the diagnosis of low-risk patients is reliable.

**Table 2:** Performance metrics for 8-variables models in predicting 30-days death in validation cohort

Model	XGB	LR
AUC	0.824	0.746
F1 Score	0.786	0.715
F2 Score	0.629	0.586
Negative Predictive Value	0.904	0.885

*Abbreviations*: *XGB* eXtreme gradient boosting, *LR* logistic regression, *AUC* area under the curve

We found that the AUC of the simplified XGBoost model was higher in predicting main outcomes and had good stability. So we compared the prediction probability of all-cause mortality of the simplified XGBoost model with the sPESI score in the validation cohort. The NRI and IDI results of the simplified XGBoost model compared with sPESI in the validation cohort are shown in Table 3. The prediction efficiency of the simplified XGBoost models was higher than the sPESI in predicting primary outcomes (NRI (Categorical) and IDI > 0, *P* < 0.001). We then defined patients in the validation group of whom the simplified XGBoost prediction probability of 30-days death was lower than 20% as the Low-risk group. The intermediate-risk group was defined as prediction probability between 20 to 40%. And the rest were the high-risk group. The outcome in each risk group defined by simplified XGBoost prediction probability is shown in Table 4. Patients’ all-cause mortality within 30 days, in-ICU mortality, in-Hospital mortality, and AKI grades increased as risk levels increased (*P* < 0.001). This result had a common trend with ESC model, SOFA and SAPSII (*P* < 0.001). Likewise, ICU stay length increased with increased risk, however, there was no such trend in hospital stay length. Moreover, for patients in the low-risk group, the occurrence of AKI was mostly stage 1–2, while for patients in the high-risk group was mostly stage 3.

**Table 3:** NRI and IDI results of simplified XGB models compared with sPESI in validation cohort

Items	Compared to sPESI	*P* value
30-days Death		
NRI (Categorical) [95% CI]	0.287 [ 0.0697—0.5043]	0.010
NRI (Continuous) [95% CI]	0.232 [ -0.0347—0.4987]	0.088
IDI [95% CI]	0.1421 [0.0684—0.2158]	< 0.001

The top 8 relative more important variables were chosen to build simplified models. Cut-off value of 30-days Mortality for categorical NRI was 0.2, 0.4 and 1

*Abbreviations*: *NRI* net reclassification improvement, *IDI* integrated discrimination improvement, *sPESI* simplified pulmonary embolism severity index, *AKI* acute kidney injury

**Table 4:** Outcome in each risk group defined by simplified XGB 30-days death prediction probability in validation cohort

Items	Low Risk (*N* = 290)	Intermediate Risk (*N* = 26)	High Risk (*N* = 52)	*P* Value
30-days Death	26 (9.0%)	11 (42.3%)	28 (53.8%)	< 0.001
In-Hospital Death	29 (10.0%)	11 (42.3%)	29 (55.8%)	< 0.001
In-ICU Death	14 (4.8%)	7 (26.9%)	25 (48.1%)	< 0.001
AKI	165 (56.9%)	22 (84.6%)	44 (84.6%)	< 0.001
AKI Stage				< 0.001
0	125 (43.1%)	4 (15.4%)	8 (15.4%)	
1	27 (9.3%)	1 (3.8%)	2 (3.8%)	
2	86 (29.7%)	10 (38.5%)	12 (23.1%)	
3	52 (17.9%)	11 (42.3%)	30 (57.7%)	
Hospital Stay Length	8.05 [4.83, 15.56]	9.52 [5.24, 15.74]	7.82 [3.13, 18.29]	0.606
ICU Stay Length	2.09 [1.06, 4.71]	2.68 [1.85, 6.64]	3.20 [1.40, 8.64]	0.032
sPESI score	2.00 [1.00, 3.00]	2.00 [1.00, 3.00]	3.00 [2.00, 4.00]	< 0.001
ESC model				< 0.001
High	74 (25.5%)	12 (46.2%)	40 (76.9%)	
Intermediate	193 (66.6%)	13 (50.0%)	11 (21.2%)	
Low	23 (7.9%)	1 (3.8%)	1 (1.9%)	
SOFA	2.50 [1.00, 5.00]	5.50 [4.25, 6.75]	9.00 [7.00, 13.25]	< 0.001
SAPSII	30.00 [23.00, 39.00]	43.00 [33.25, 48.75]	52.50 [41.00, 63.75]	< 0.001

The top 8 relative more important variables were chosen to build simplified models. Low-risk group was defined as patients whose XGB prediction ability lower than 20%, Intermediate-risk group was defined as patients whose XGB prediction ability between 20 to 40%, High-risk group was defined as patients whose XGB prediction ability higher than 40%. Categorical variables are expressed as percentages, compared with chi-square tests, continuous variables were represented by the median with interquartile range (IQR) and compared using the Kruskal–Wallis test. A two-sided *P* < 0.05 was considered statistically significant

*Abbreviations*: *XGB* eXtreme gradient boosting, *AKI* acute kidney injury, *sPESI* simplified pulmonary embolism severity index, *ESC* European society of cardiology, *SAPSII* simplified acute physiology score II, *SOFA* sequential organ failure assessment, *ICU* intensive care unit

**Fig. 3:**
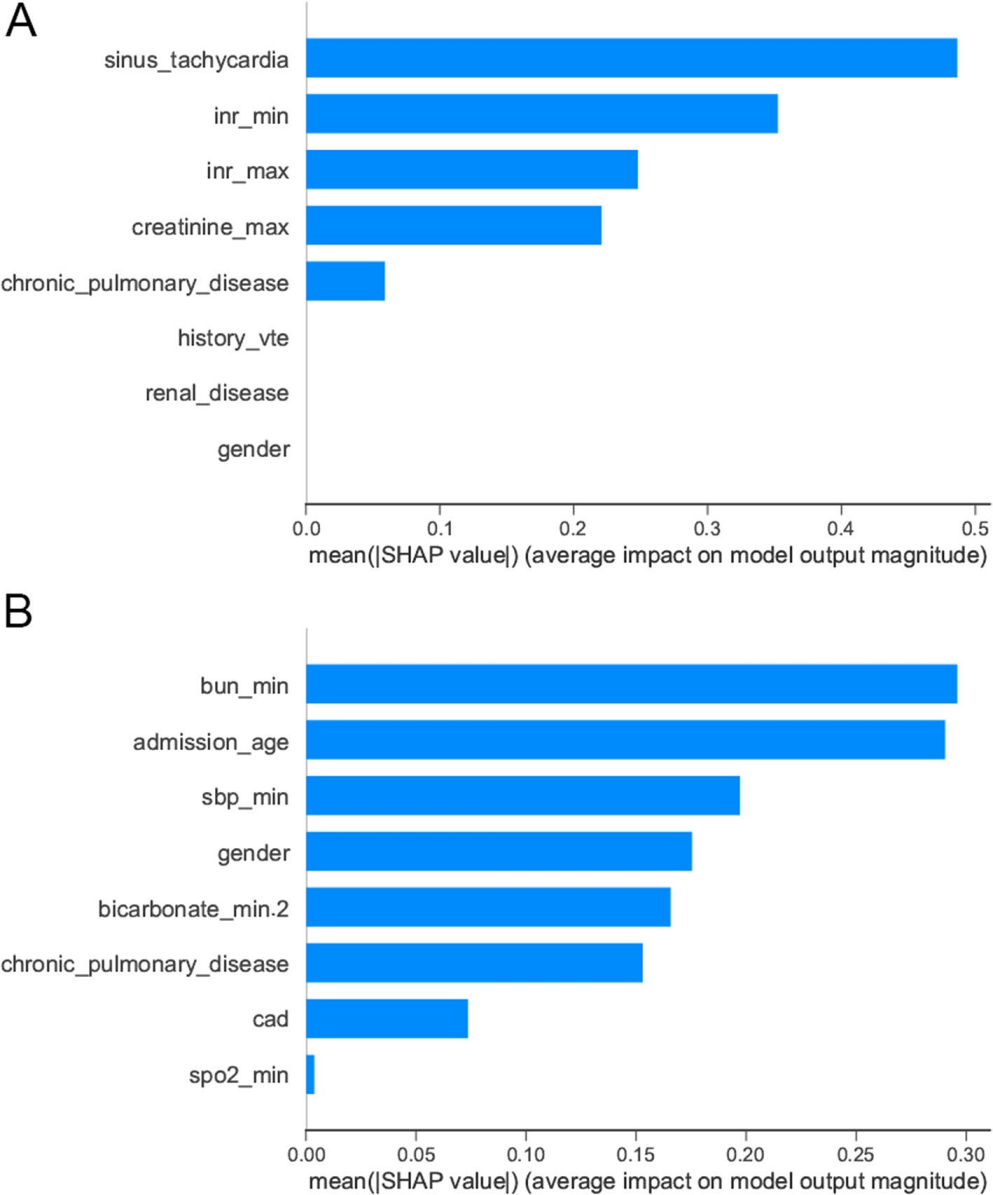
Bar graph consisting of average SHAP value for each feature. The mean absolute Shapley values are measured as feature importance. A feature is considered to be “important” if its mean absolute Shapley value is high; a feature is considered to be “unimportant” if its mean absolute Shapley value is low or zero

To characterize the crucial characteristics that affect mortality risk in ML model, we plotted bar graph consisting of average SHAP value for each feature (Fig. 3). The results showed that the marginal contribution of sinus tachycardia, INR-max and INR-min to the XGBoost model were the highest, while blood urea nitrogen (minimum), age and systolic blood pressure (minimum) contributed more to the LR model.

**Fig. 4:**
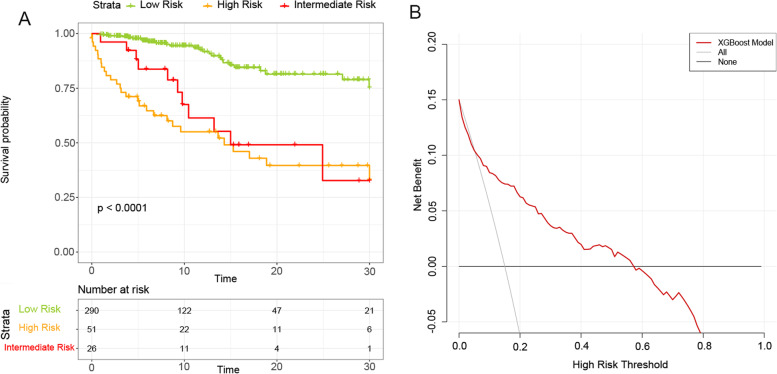
Survival curves within 30 days for each group in validation cohort and decision curve analysis based on the XGBoost model. **A** Survival curves within 30 days for each group in validation cohort; **B** Decision curve analysis based of XGBoost model

Patients in the validation cohort were stratified based on the XGBoost model and plotted survival curves within one month for each group (Fig. 4A). The results showed that there were differences in survival between different groups (*P* < 0.001), and the short-term mortality of low-risk patients was much lower than that of intermediate and high-risk patients. After We had further performed decision curve analysis (DCA) on XGBoost model, patients using this model for risk stratification obtained more net benefit, with threshold set approximately between 0.1 and 0.6 (Fig. 4B).

## Discussion

In this study, we used data from 1229 patients to develop and validate models with different ML algorithms. XGBoost was chosen to construct the finally model. After training and validation, we found that our finally model had good predictive power for the primary outcome. The simplified XGB model performed well in predicting death events within one month with a AUC of 0.82 (95% CI: 0.78–0.87). Based on risk deciles and calibration plots of ML models, patients were grouped into different risk levels and the new classification systems based on XGB could accurately predict low-risk of mortality, and had high consistency with acknowledged risk scores. Moreover, compared with sPESI, the prediction efficiency of the simplified XGBoost models was higher in predicting 30-days Death (NRI (Categorical) and IDI > 0, *P* < 0.001).

Many models have already been developed for the prognosis of PE. Massimo Cugno’s team used the 2014 ESC model to predict early mortality in PE patients [21]. Anthony J. Weekes et al. developed a tool named Pulmonary embolism short-term clinical outcomes risk estimation (PE-SCORE) to assess short-term clinical outcomes in patients with PE [22]. Recently, much more popular models are PESI, sPESI, Angriman, etc. Besides, there are many models that combine them [23–25]. However, no clear model has been especially built for risk and heterogeneity classification of critical PE patients who need ICU admission.

ML is the learning of data that captures high-dimensional, non-linear relationships between clinical features and makes predictions [15]. In ML, algorithms learn patterns from data without being explicitly programmed with pre-specified rules [26, 27]. Compared with traditional modeling methods, ML has much more advantages in processing real-world data, such as (i) ML can process high-dimensional, complex variables from clinical practice; (ii) ML has better generalization and accuracy [28]. ML has been widely recognized and applied in many fields of medicine in recent years. For example, ADB model has been used to predict adverse events in acute coronary syndromes [29], RF model was used for risk assessment of delayed graft function in kidney transplantation [30]. Based on the advantages of ML, we developed several different models to predict the prognosis of patients with critical PE. Our aim is to classify patients with PE. For high-risk patients, we should intervene as early as possible to prevent the occurrence of the worse outcomes, especially the occurrence of renal failure and death. As for low-risk patients, it is our responsibility to reduce their ICU stay length and make the precious medical resources in the ICU reasonably allocated. XGBoost, a decision-tree-based algorithm, can automatically learn the splitting direction for its missing data. The underlying tree structure of XGB is the Classification and Regression Tree (CART). This is a parameter-based algorithm that is used to train the model after dividing the dataset. Because of its high precision, flexibility and regularization, it is widely used in the field of medical research [31]. LR is a ML method used to solve a binary classification problem for estimating the likelihood of something. We can see the impact of different features on the final outcome by the weight of the features, therefore the interpretability of LR is high [19, 32].

The 8 factors that have the greatest impact on the outcome based on different algorithms for each model are presented as radar graphs. INR (maximum and minimum), gender, as well as sinus-tachycardia had the greatest impact on the prognosis of patients in XGBoost model. Anticoagulation therapy has long been considered the cornerstone of treatment for PE, patients should receive anticoagulant therapy regardless of risk stratification [6]. Insufficient anticoagulant dose and time are associated with poor prognosis, while excessive anticoagulant will increase the risk of bleeding and affect the prognosis of patient [6, 33]. The rise or fall of INR reflects insufficient or excessive anticoagulation for PE, thus predicting the patient's prognosis. Male sex and tachycardia have been proven as an important prognostic indicator of pulmonary embolism and has been included in previous prognostic scoring systems, such as PESI, which is consistent with the included predictive factors in our model [5, 25]. A rise in serum creatinine always indicates the occurrence of AKI, previous studies show that patients with critical PE are more likely to develop AKI [34, 35]. Mechanisms that may cause AKI in PE include the renal hypoperfusion, comorbidities of critical PE patients as well as respiratory failure and anemia caused by PE [35, 36]. Factors such as chronic pulmonary disease, renal disease, and history of VTE are also important factors contributing to increased patient mortality [35, 37]. Patients with renal disease were more likely to develop AKI, while the hazard ratio increased to 1.8 (95% CI: 1.2–2.7) for PE patients with chronic lung disease [38]. Therefore, we should not only concentrate on the treatment of thrombosis, but also pay attention to the underlying diseases of patients and improve the function of various organs, so as to improve the prognosis. Although the variables screened by each model are not the same as the clinical risk factors, they still have certain guiding significance for our judgment of the patient's condition [37]. In addition, hemoglobin levels and anemia have been shown to be associated with all-cause death, recurrent, and major bleeding in patients with acute coronary syndromes (ACS), which are considered an important prognostic factor and are included in the existing prognostic model of ACS, but were not included in our model [39, 40]. This may be because the main cause of death in PE is hemodynamic deterioration or respiratory failure, rather than bleeding.

To further test the validity of the model, we calculated NRI for the three primary outcomes compared with sPESI score using XGBoost model. We can see that the accuracy of the simplified XGBoost models improved with different cutoff values (NRI (Categorical) > 1, *P* < 0.001). At the same time, we calculated IDI to investigate the overall improvement of the simplified XGBoost model. The results also showed that our model was overall better than sPESI score (IDI > 1, *P* < 0.001). We entered baseline data, vital signs, and laboratory data from patients within 24 h, complications, treatment information and severity scores after admission into a simplified XGB model for analysis. According to the analysis results, the prognosis of patients could be stratified. We divided patients into the low-risk group, intermediate-risk group, and high-risk group using predicted probabilities of 20% and 40% as cut-off points. We could see that mortality was higher in patients within higher risk groups, whether 30-days Death, in-ICU death or in-Hospital death. Notably, the 30-day mortality rate in the low-risk group was 9.0%, with the In-ICU mortality was 4.8%, significantly lower than in the intermediate-risk group and high-risk group, and patients in the low-risk group had significantly lower occurrence than those in the other two groups in incidence of renal failure, or grade of renal failure. While no significant difference in the 30-day mortality rate (42.3%, 53.8%) and AKI incidence (84.6%, 84.6%) between the intermediate-risk group and the high-risk group. Thus, patients identified as low risk by the model are safe enough, and should be considered for transfer out of the ICU for further treatment, while intermediate-risk patients should continue to be monitored in the ICU. Similarly, the negative predictive value of our model is as high as 0.904 (Table 2), which also confirms low-risk patients are safe enough.

To the best of our knowledge, this study is the first to use the ML algorithm to develop models for predicting the prognosis of patients with critical PE. And our models have better predictive ability than the other models or scores. Moreover, our model can also stratify the risk of AKI in PE patients, which previous models cannot do. When our models are refined, triage for critical PE patients in ICU can be improved clinically. Reasonable allocation of ICU resources can effectively improve patient outcomes, survival length and quality of life.

The Management Strategy and Prognosis of Pulmonary Embolism Registry (MAPPET) registry reported that an overall mortality rate of 1001 patients with PE is 29% [41]. While in our cohort used to build models, the 30-days mortality was about 16.2%. This may be the reflection of medical progress. Advanced examination equipments, assessment systems and treatment levels significantly reduce mortality of PE patinets. However, the mortality is still high. High-efficiency assessment systems are needed. We used ML to predict the prognosis of critical PE in order to explore a new assessment system. A good assessment system can identify high-risk patients and allow us to intervene promptly and improve prognosis. It can also help us identify low-risk patients and allow patients to discharge early so as to avoid wasting ICU resources. Thus, reasonable risk grading is beneficial to triage, so that we can reasonably allocate medical resources and save more life.

However, this study has limitations. Firstly, this is a retrospective study based on the MIMIC-IV database. The MIMIC-IV database is not used specifically for modeling, some important indicators related to PE are not completely collected at the time of data collection. Such as recent surgery, D-dimer and so on [37]. We hope to add these well-established risk factors to refine our model in future studies. Perhaps the database of special diseases in different hospitals can help us solve this problem. Secondly, although our model performed well in prediction. ROC curves of XGBoost model performed well for the primary outcomes. Our modeling still requires external validation from multiple different centers to revise our model and improve its generalizability. Third, our models have black-box problems, which make the model interpretability and transparency limited. The black-box problem is also a major problem that limits the practical application of ML in clinical practice, so improving the interpretability of the model is the key to this problem [42]. We should also actively explore new models to enhance interpretability and thus better apply ML in clinical practice.

## Conclusion

In this study, we used ML algorithms to develop and validate models for predicting 30-Days mortality of AKI for critical PE admitted to ICU. ML models helped accurately predict the occurrence of 30-Days mortality, which could further be used to reduce the burden of ICU stay and decrease the mortality, increase the quality of patients’ life in the clinic.

## Supplementary Information

**Additional file 1: Supplementary Table 1.** Variables used for selecting and model fitting. **Supplemental Figure 1.** Survival Curves in derivation and validation cohort. **Supplemental Figure 2.** Radar plot for 30-days mortality. **Supplemental Figure 3.** Risk of outcome in derivation cohort according to deciles of event probability based on Top 8 variables models. **Supplemental Figure 4.** Calibration curve based on Top 8 variables models in derivation cohort.

## Data Availability

The data that support the findings of this study are available from https://mimic.mit.edu/, but restrictions apply to the availability of these data, which were used under license for the current study, and so are not publicly available. Data are however available from the Kai Huang upon reasonable request and with permission of Medical Information Mart for Intensive Care.

## Abbreviations

BMI: Body mass index
DVT: Deep vein thrombosis
VTE: Venous thromboembolism
sPESI: Simplified pulmonary embolism severity index
ESC: European society of cardiology
SAPSII: Simplified acute physiology score II
SOFA: Sequential organ failure assessment
ICU: Intensive care unit
AKI: Acute kidney injury
NRI: Net reclassification improvement
IDI: Integrated discrimination improvement
XGB: EXtreme gradient boosting
ROC: Receiver operating characteristic curve
AUC: Area under the curve
RF: Random forest
MLP: Multilayer perceptron
ADB: Adaptive boosting
LR: Logistic regression
ML: Machine learning
XGB: EXtreme gradient boosting
MLP: Multilayer perceptron
MLP: Multilayer perceptron
