# Supplementary material for: Machine learning-based models for predicting mortality and acute kidney injury in critical pulmonary embolism
Source: BMC Cardiovasc Disord. 2023 Aug 2;23:385. doi: 10.1186/s12872-023-03363-z (PMC10399014; doi:10.1186/s12872-023-03363-z)

**Machine Learning-based Models for Predicting Mortality and Acute Kidney Injury in Critical Pulmonary Embolism**

**Index**

**Supplemental Figures and Tables**

[Supplementary Table 1. Variables used for selecting and model fitting 2](#_Toc135743746)

[Supplemental Figure 1 Survival Curves in derivation and validation cohort 6](#_Toc135743747)

[Supplemental Figure 2 Radar plot for 30-days mortality 7](#_Toc135743748)

[Supplemental Figure 3 Risk of outcome in derivation cohort according to deciles of event probability based on Top 8 variables models 8](#_Toc135743749)

[Supplemental Figure 4 Calibration curve based on Top 8 variables models in derivation cohort 9](#_Toc135743750)

# Supplementary Table 1. Variables used for selecting and model fitting

| Variables | Derivation Cohort  (N = 860) | Validation Cohort  (N = 369) | P Value | Missing Value |
| --- | --- | --- | --- | --- |
| Male | 439 (51.0%) | 179 (48.5%) | 0.451 | 0 |
| Age | 67.46 [55.88, 78.93] | 65.86 [54.61, 75.67] | 0.034 | 0 |
| Proximal DVT | 114 (13.3%) | 63 (17.1%) | 0.097 | 0 |
| VTE History | 43 (5.0%) | 19 (5.1%) | 1 | 0 |
| Hematocrit (min) | 31.40 [27.00, 36.55] | 32.50 [27.40, 36.90] | 0.235 | 5 |
| Hematocrit (max) | 35.00 [30.70, 40.20] | 36.30 [31.40, 40.80] | 0.144 | 5 |
| Hemoglobin (min) | 10.40 [8.70, 12.00] | 10.75 [8.80, 12.22] | 0.194 | 6 |
| Hemoglobin (max) | 11.50 [9.90, 13.30] | 11.90 [10.00, 13.50] | 0.137 | 6 |
| Platelets (min) | 198.00 [138.00, 265.00] | 189.00 [144.00, 254.00] | 0.577 | 5 |
| Platelets (max) | 229.00 [167.50, 301.00] | 222.00 [171.00, 293.00] | 0.656 | 5 |
| WBC (min) | 9.60 [7.10, 12.70] | 9.50 [7.20, 12.72] | 0.956 | 6 |
| WBC (max) | 12.30 [9.10, 16.35] | 11.90 [9.00, 16.80] | 0.567 | 6 |
| Anion Gap (min) | 13.00 [11.00, 15.00] | 13.00 [11.00, 15.00] | 0.51 | 7 |
| Anion Gap (max) | 16.00 [14.00, 19.00] | 16.00 [14.00, 19.00] | 0.864 | 7 |
| Bicarbonate (min) | 22.00 [19.00, 25.00] | 22.00 [19.00, 25.00] | 0.318 | 7 |
| Bicarbonate (max) | 24.00 [22.00, 27.00] | 24.00 [22.00, 27.00] | 0.802 | 7 |
| BUN (min) | 16.00 [12.00, 24.00] | 16.00 [11.00, 26.00] | 0.91 | 6 |
| BUN (max) | 19.00 [14.00, 28.75] | 19.00 [14.00, 30.00] | 0.767 | 6 |
| Calcium (min) | 8.30 [7.70, 8.70] | 8.20 [7.70, 8.70] | 0.497 | 42 |
| Calcium (max) | 8.60 [8.10, 9.10] | 8.60 [8.20, 9.00] | 0.705 | 42 |
| Chloride (min) | 102.00 [98.00, 105.00] | 102.00 [99.00, 105.00] | 0.918 | 7 |
| Chloride (max) | 105.00 [101.00, 108.00] | 105.00 [101.00, 108.00] | 0.69 | 7 |
| Creatinine (min) | 0.80 [0.60, 1.10] | 0.90 [0.60, 1.10] | 0.293 | 5 |
| Creatinine (max) | 1.00 [0.70, 1.30] | 1.00 [0.70, 1.30] | 0.366 | 5 |
| Sodium (min) | 137.00 [135.00, 140.00] | 137.00 [135.00, 140.00] | 0.536 | 7 |
| Sodium (max) | 140.00 [137.00, 143.00] | 140.00 [137.00, 142.00] | 0.372 | 7 |
| Potassium (min) | 3.80 [3.50, 4.20] | 3.90 [3.50, 4.20] | 0.636 | 7 |
| Potassium (max) | 4.30 [4.00, 4.80] | 4.40 [4.00, 4.90] | 0.508 | 7 |
| Heart Rate (min) | 76.00 [65.00, 88.00] | 76.00 [64.00, 88.00] | 0.641 | 3 |
| Heart Rate (max) | 109.00 [95.00, 124.00] | 109.00 [94.00, 123.00] | 0.515 | 3 |
| SBP (min) | 91.50 [82.00, 103.00] | 92.00 [82.00, 103.00] | 0.524 | 5 |
| SBP (max) | 145.00 [131.00, 158.00] | 145.00 [131.75, 159.00] | 0.786 | 5 |
| DBP (min) | 49.00 [41.00, 57.00] | 49.00 [41.75, 57.00] | 0.984 | 5 |
| DBP (max) | 90.00 [79.75, 102.00] | 88.50 [79.00, 104.00] | 0.853 | 5 |
| MBP (min) | 61.00 [53.00, 69.00] | 61.00 [53.00, 69.00] | 0.587 | 3 |
| MBP (max) | 102.00 [92.50, 115.00] | 102.00 [92.00, 116.00] | 0.962 | 3 |
| INR (min) | 1.20 [1.10, 1.40] | 1.20 [1.10, 1.40] | 0.691 | 62 |
| INR (max) | 1.30 [1.20, 1.60] | 1.30 [1.20, 1.60] | 0.732 | 62 |
| PT (min) | 13.50 [12.30, 15.28] | 13.40 [12.30, 15.00] | 0.673 | 61 |
| PT (max) | 14.60 [13.10, 17.30] | 14.45 [13.00, 17.65] | 0.656 | 61 |
| PTT (min) | 30.50 [26.40, 42.15] | 30.30 [26.30, 45.30] | 0.609 | 52 |
| PTT (max) | 69.40 [31.10, 142.45] | 66.40 [31.90, 127.03] | 0.632 | 52 |
| Respiratory Rate (min) | 14.00 [11.00, 16.00] | 14.00 [11.00, 16.00] | 0.755 | 3 |
| Respiratory Rate (max) | 29.00 [25.00, 33.00] | 29.00 [25.00, 34.00] | 0.542 | 3 |
| Temperature (min) | 36.44 [36.11, 36.67] | 36.50 [36.22, 36.67] | 0.717 | 27 |
| Temperature (max) | 37.22 [36.94, 37.70] | 37.17 [36.94, 37.61] | 0.799 | 27 |
| SpO2 (min) | 92.00 [89.00, 94.00] | 92.00 [89.00, 94.00] | 0.97 | 3 |
| SpO2 (max) | 100.00 [99.00, 100.00] | 100.00 [99.00, 100.00] | 0.11 | 3 |
| Glucose (min) | 108.00 [94.00, 129.00] | 109.00 [94.00, 129.00] | 0.925 | 25 |
| Glucose (max) | 148.00 [117.00, 200.25] | 142.50 [117.00, 198.50] | 0.56 | 25 |
| Septicemia | 79 (9.2%) | 35 (9.5%) | 0.953 | 0 |
| Congestive Heart Failure | 202 (23.5%) | 100 (27.1%) | 0.202 | 0 |
| Hypertension | 426 (49.5%) | 172 (46.6%) | 0.38 | 0 |
| Stroke | 40 (4.7%) | 24 (6.5%) | 0.23 | 0 |
| Atrial Fibrillation | 208 (24.2%) | 85 (23.0%) | 0.718 | 0 |
| Coronary Artery Disease | 121 (14.1%) | 46 (12.5%) | 0.508 | 0 |
| COPD | 186 (21.6%) | 72 (19.5%) | 0.448 | 0 |
| Myocardial Infarct | 105 (12.2%) | 43 (11.7%) | 0.858 | 0 |
| Peripheral Vascular Disease | 67 (7.8%) | 24 (6.5%) | 0.502 | 0 |
| Cerebrovascular Disease | 117 (13.6%) | 48 (13.0%) | 0.849 | 0 |
| Dementia | 28 (3.3%) | 14 (3.8%) | 0.761 | 0 |
| Chronic pulmonary disease | 245 (28.5%) | 110 (29.8%) | 0.689 | 0 |
| Rheumatic Disease | 38 (4.4%) | 10 (2.7%) | 0.209 | 0 |
| Peptic Ulcer Disease | 23 (2.7%) | 12 (3.3%) | 0.711 | 0 |
| Mild Liver Disease | 89 (10.3%) | 38 (10.3%) | 1 | 0 |
| Severe Liver Disease | 16 (1.9%) | 4 (1.1%) | 0.459 | 0 |
| Diabetes without Critical Complication | 177 (20.6%) | 68 (18.4%) | 0.431 | 0 |
| Diabetes with Critical Complication | 49 (5.7%) | 29 (7.9%) | 0.195 | 0 |
| Paraplegia | 44 (5.1%) | 20 (5.4%) | 0.937 | 0 |
| Renal Disease | 114 (13.3%) | 53 (14.4%) | 0.668 | 0 |
| Malignant Cancer | 212 (24.7%) | 91 (24.7%) | 1 | 0 |
| Metastatic Solid Tumor | 155 (18.0%) | 57 (15.4%) | 0.311 | 0 |
| Hemodynamic Instability | 304 (35.3%) | 127 (34.4%) | 0.804 | 0 |
| Urine Output in First Day | 1280.00 [791.25, 2043.75] | 1305.00 [800.00, 2020.00] | 0.75 | 37 |
| Ventilation |  |  | 0.834 | 0 |
| None | 117 (13.6%) | 55 (14.9%) |  |  |
| Non-Invasive | 473 (55.0%) | 200 (54.2%) |  |  |
| Invasive | 270 (31.4%) | 114 (30.9%) |  |  |
| Invasive Line | 469 (54.5%) | 208 (56.4%) | 0.596 | 0 |

Categorical variables were expressed as percentages, compared with chi-square tests, continuous variables were represented by the median with interquartile range (IQR) and compared using the Kruskal–Wallis test. A two-sided P < 0.05 was considered statistically significant.

Abbreviation: DVT, deep vein thrombosis; VTE, venous thromboembolism; WBC, white blood cell; BUN, blood urea nitrogen; SBP, systolic blood pressure; DBP, diastolic blood pressure; MBP, mean blood pressure; INR, international normalized ratio; PT, prothrombin time; PTT, partial thromboplastin time; SpO2, percutaneous oxygen saturation; COPD, chronic obstruction pulmonary disease; ICU, intensive care unit.

# Supplemental Figure 1 Survival Curves in derivation and validation cohort


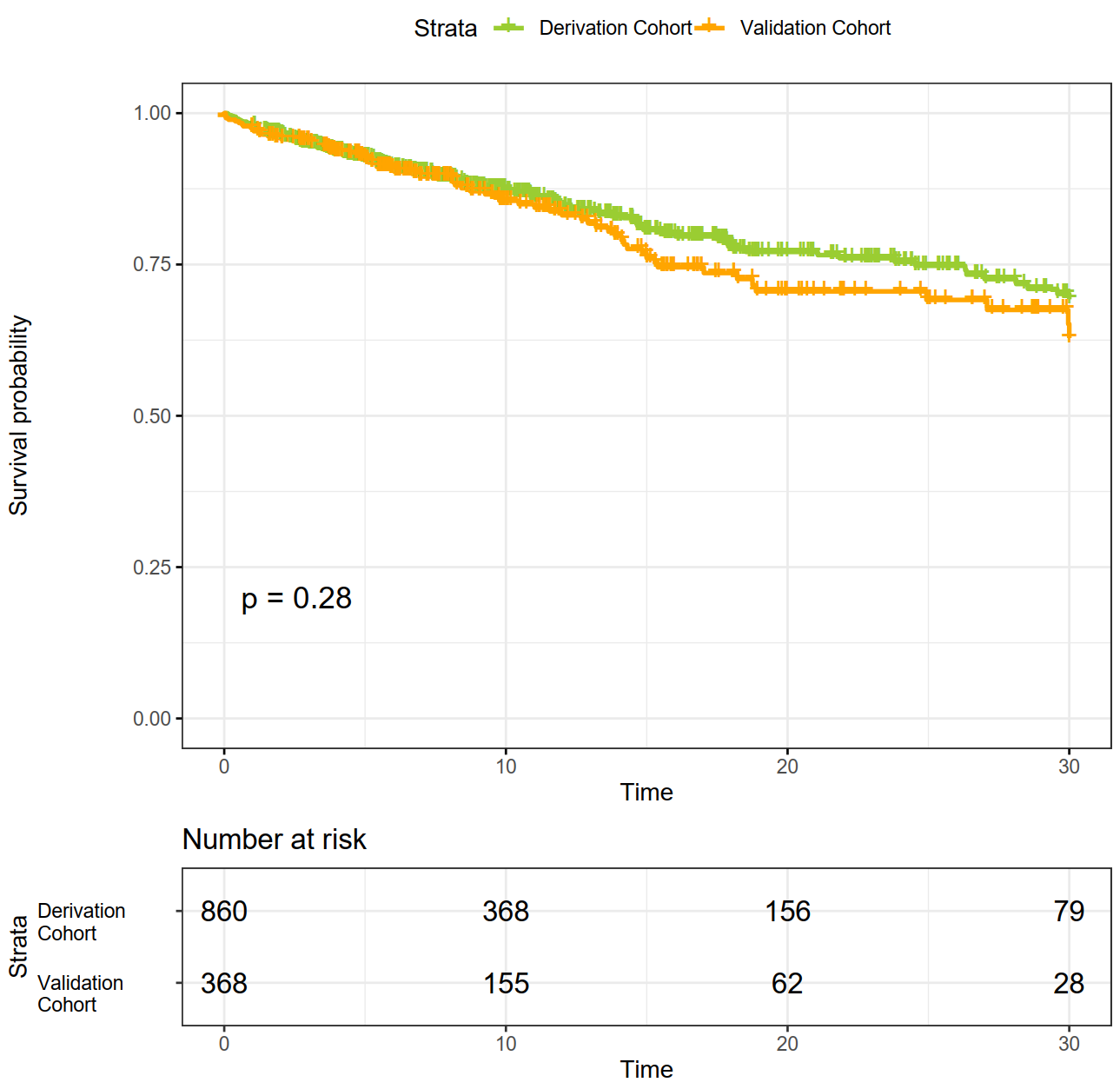


# Supplemental Figure 2 Radar plot for 30-days mortality


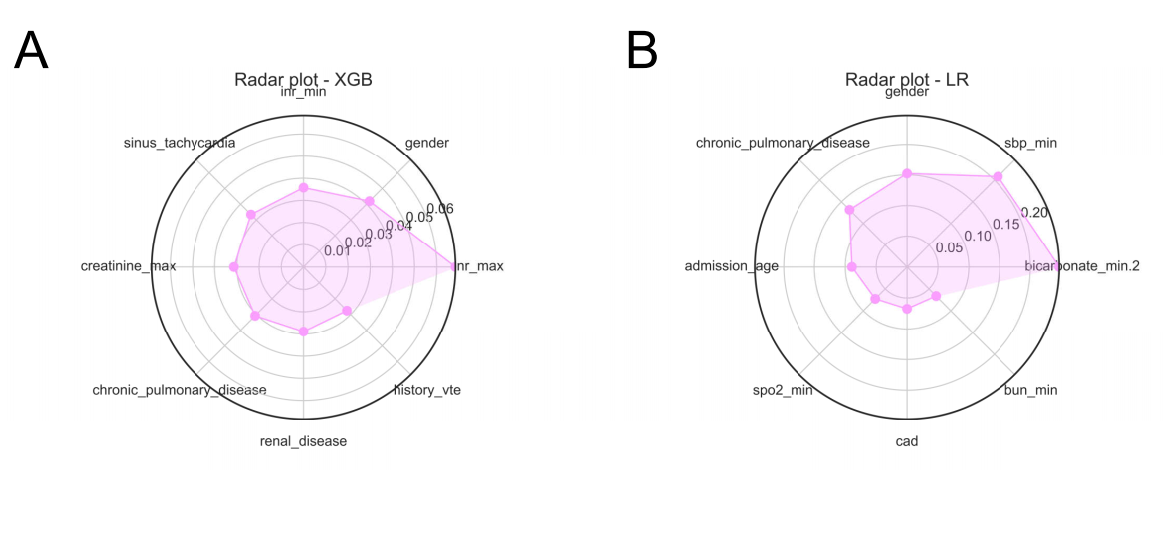


Higher value means more importance of the features determined by different ML algorithms.

Abbreviation: XGB, eXtreme gradient boosting; LR, logistic regression.

# Supplemental Figure 3 Risk of outcome in derivation cohort according to deciles of event probability based on Top 8 variables models


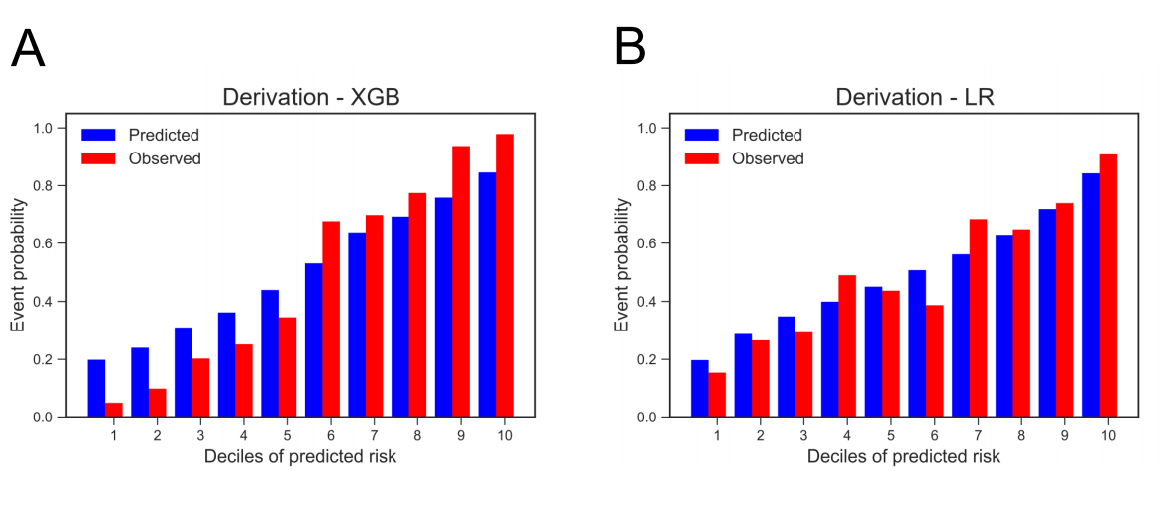


# Supplemental Figure 4 Calibration curve based on Top 8 variables models in derivation cohort


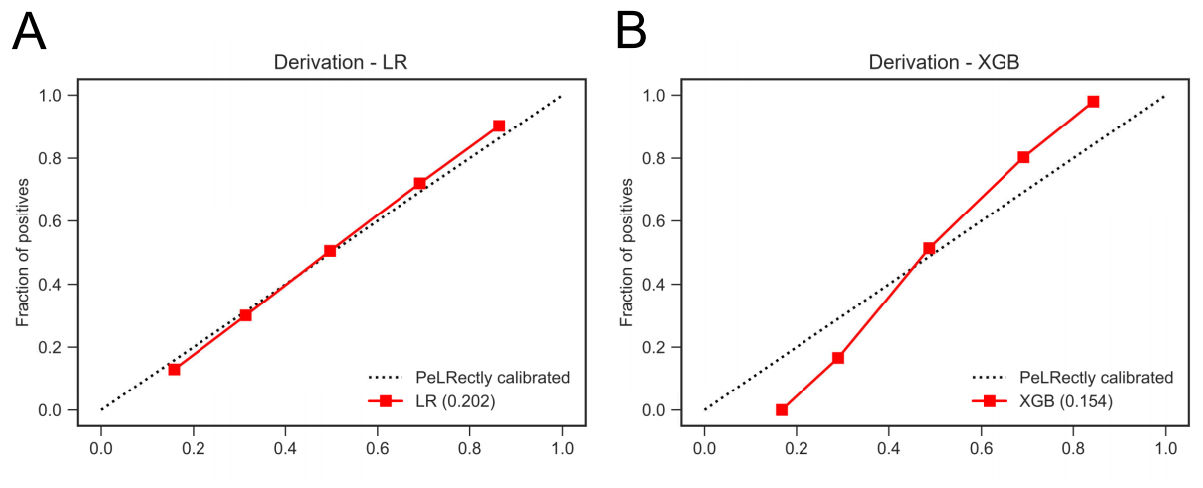

Supplement: Supplementary file 1 — Additional file 1: Supplementary Table 1. Variables used for selecting and model fitting. Supplemental Figure 1. Survival Curves in derivation and validation cohort. Supplemental Figure 2. Radar plot for 30-days mortality. Supplemental Figure 3. Risk of outcome in derivation cohort according to deciles of event probability based on Top 8 variables models. Supplemental Figure 4. Calibration curve based on Top 8 variables models in derivation cohort. [file 12872_2023_3363_MOESM1_ESM.docx]
